# Domino Multicomponent Approach for the Synthesis of Functionalized Spiro-Indeno[1,2-*b*]quinoxaline Heterocyclic Hybrids and Their Antimicrobial Activity, Synergistic Effect and Molecular Docking Simulation

**DOI:** 10.3390/molecules24101962

**Published:** 2019-05-22

**Authors:** Abdulrahman I. Almansour, Natarajan Arumugam, Raju Suresh Kumar, Dhaifallah M. Al-thamili, Govindasami Periyasami, Karuppiah Ponmurugan, Naif Abdullah Al-Dhabi, Karthikeyan Perumal, Dhanaraj Premnath

**Affiliations:** 1 Department of Chemistry, College of Science, King Saud University, P.O. Box 2455, Riyadh 11451, Saudi Arabia; almansor@ksu.edu.sa (A.I.A.); sraju@ksu.edu.sa (R.S.K.); daife54321@hotmail.com (D.M.A.); pkandhan@ksu.edu.sa (G.P.); 2 Department of Botany and Microbiology, College of Science, King Saud University, Riyadh 11451, Saudi Arabia; pkaruppiah@ksu.edu.sa (K.P.); naldhabi@ksu.edu.sa (N.A.A.-D.); 3 Department of Chemistry and Biochemistry, The Ohio State University, 151 W. Woodruff Ave, Columbus, OH 43210, USA; pkarthikjaya@gmail.com; 4 Department of Bioscience and Technology, Karunya Institute of Technology and Science, Branch of Bioinformatics, School of Agriculture and Biosciences, Karunya Nagar, Coimbatore-641114, India; prems.bioinfo@gmail.com

**Keywords:** domino multicomponent reaction, dispiropyrrolidine, indeno[1,2-*b*]quinoxaline, docking studies, antimicrobial activity, synergistic effect

## Abstract

An expedient synthesis of hitherto unexplored novel hybrid heterocycles comprising dispiropyrrolidine, *N*-styrylpiperidone and indeno[1,2-*b*]quinoxaline units has been developed via domino multicomponent 1,3-dipolar cycloaddition strategy employing a new class of azomethine ylide in ionic liquid, 1-butyl-3-methylimidazolium bromide. This domino protocol involves, 1,3-dipolar cycloaddition and concomitant enamine reaction affording the dispiropyrrolidine tethered *N*-styrylpiperidone hybrid heterocycles in moderate to good yield in a single step. These compounds were evaluated for their antimicrobial activity against bacterial and fungal pathogens, therein compounds **8f**, **8h**, and **8l** displayed significant activity against tested microbial pathogens. The synergistic effect revealed that the combination of compound **8h** with streptomycin and vancomycin exhibited potent synergistic activity against *E. coli* ATCC 25922. In addition, molecular docking simulation has also been studied for the most active compound.

## 1. Introduction

**Figure 1:**
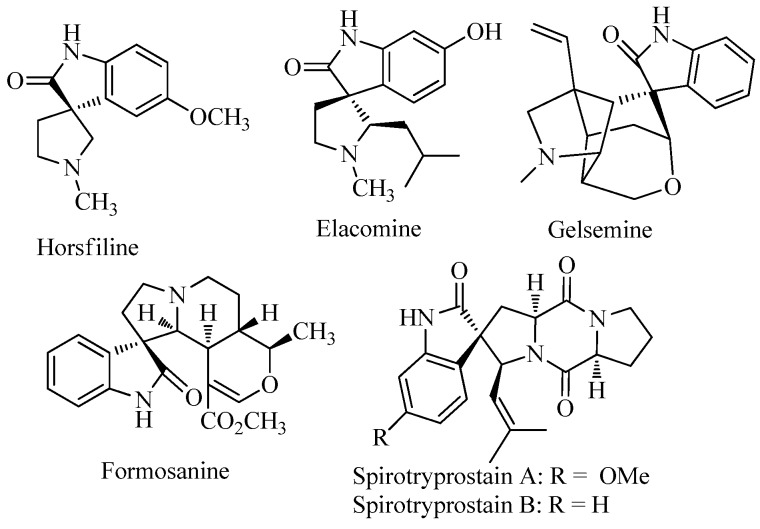
Biologically important spiropyrrolidine heterocycles.

The increasing emergence of drug resistance, intractable pathogenic microorganisms, and newly arising pathogens have become a serious and challenging problem for human health. This situation stimulates an urgent need to develop novel antimicrobial agents with completely different chemical structures possibly exerting different mechanisms of action than current clinical drugs [1]. In this context, spirocyclic scaffolds are very attractive for drug discovery because their inherently three-dimensional structure can achieve interactions with three-dimensional binding sites more easily than when using planar (hetero) aromatic systems as ligands. Perhaps for this reason, a large number of spirocyclic cores can be found in natural products, which have evolved to interact with proteins [2]. Among them, pyrrolidine-embedded spiroatom are present in numerous alkaloids and pharmacologically important compounds including horsfiline, elacomine, gelsemine, formosanine, the spirotryprotatins A and B (Figure 1). These compounds and many more synthetic spiropyrrolidine heterocyclic hybrids have been reported to display anticancer [3,4,5], antimycobacterial [6] anti-inflammatory, analgesic [7] antimicrobial [8], and AChE inhibition activities [9,10].

**Scheme 1:**
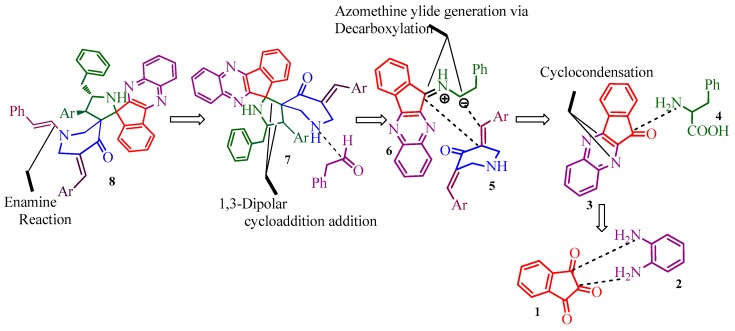
Synthetic strategy for the formation of dispiropyrrolidine heterocyclic hybrids, **8a**–**k**.

Piperidone is another important class of pharmacophore as its derivatives possess interesting biological profiles such as potential antitumor [11] and antimicrobial agents [12]. Our research group has largely been involved in the synthesis of structurally diverse novel heterocyclic hybrids comprising spiropyrrolidine, piperidone units employing 1,3-dipolar cycloaddition followed by their biological screening, which has produced various anticancer [13], antimycobacterial [14], anti-Alzheimer [15] and antimicrobial leads [16,17]. The above precedents prompted us to explore the synthesis of novel heterocyclic hybrids comprising dispiropyrrolidine, *N*-styrylpiperidinone, and indeno[1,2-*b*]quinoxaline units via domino multicomponent reaction in ionic liquid in the present investigation. Recently, several spiroheterocyclic hybrids have been synthesized in an ionic liquid medium due to their unique properties such as high thermal stability, solvating ability, recyclability, and ability to accelerate the rate of reaction that was supported by earlier reports [18,19,20]. The synthesized compounds were assayed for their antimicrobial activity against ten bacterial and five fungal pathogens and the synergistic effect of these compounds has also been investigated. The synthetic strategy for the formation of our target molecules has been described in Scheme 1.

## 2. Results and Discussion

### 2.1. Chemistry

**Scheme 2:**
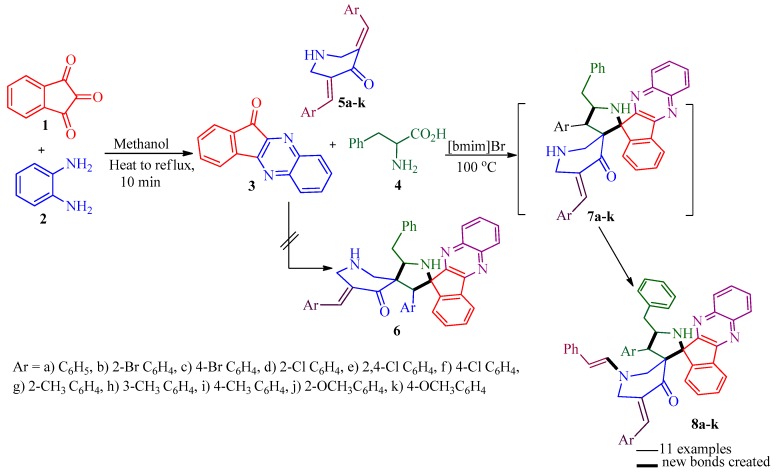
Synthesis of dispiroheterocyclic hybrids **8a**–**k** via three component domino reaction.

**Scheme 3:**
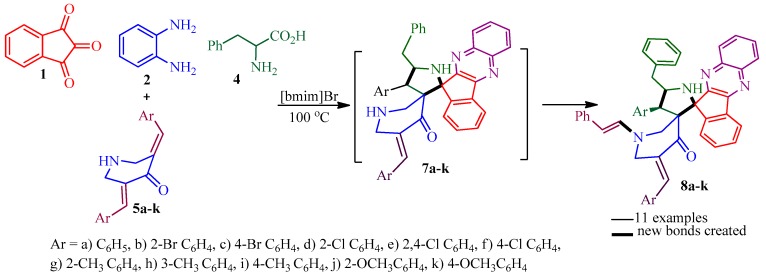
Synthesis of dispiroheterocyclic hybrids **8a**–**k** via four component domino reaction.

**Scheme 4:**
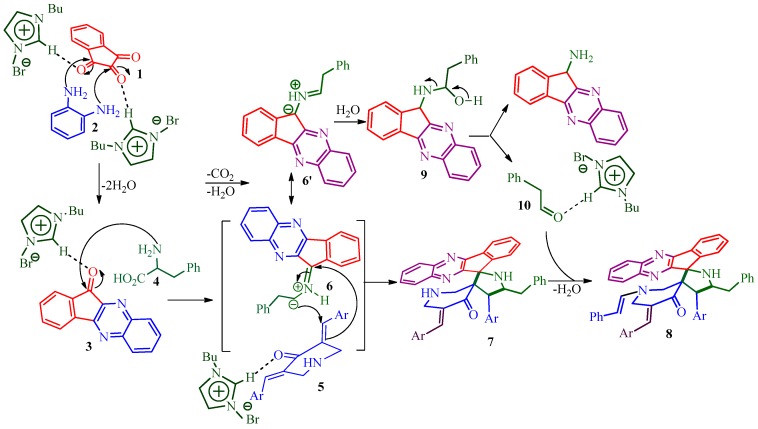
Plausible mechanism for the formation of dispiropyrrolidines heterocyclic hybrids, **8a**–**k**.

We employed a domino multicomponent 1,3-dipolar cycloaddition strategy [21,22] for the synthesis of a new class of dispiroheterocycles **8a**–**k**, the azomethine ylide employed in the present work is first of its kind and adds regioselectively to the highly functionalized dipolarophiles. Initially, a model reaction was investigated with an equimolar ratio of 3,5-dibenzylidenepiperidin-4-one **5** [18], indeno[1,2-*b*]quinoxalin-11-one **3** and l-phenylalanine **4** in refluxing methanol. Under this condition, the reaction afforded an inseparable mixture of products. Hence, in order to optimize the reaction conditions, we attempted the same reaction with different ratios of l-phenylalanine. A single product was obtained when a 2 mmol equivalent of l-phenylalanine was employed. The reaction was also performed under different solvent system including methanol, ethanol, acetonitrile, 1,4-dioxane, and the reaction afforded the product in 49,45, 42, 40, and 46% yields, respectively (Table 1). To improve the yield of product **8j**, the same reaction was also investigated in [bmim]Br at 100 °C, the reaction afforded the product in good yield (60%) after 1 h (Table 1) (Scheme 2). As a more efficient alternative, the synthesis of the target dispiroheteocyclic hybrid **8j** was also attempted through a one-pot four component cycloaddition reaction. Thus, a mixture of **1** (1 mmol), **2** (1 mmol), **4** (2 mmol), and **5j** (1 mmol) was heated at 100 °C for 1 h, furnishing the desired product in good yield (64%) in a single step without isolation of the intermediate **3** (Scheme 3). Consequently, all the subsequent reactions were performed under these optimized conditions. It is pertinent to note that the choice of l-phenylalanine for the reaction with indeno[1,2-*b*]quinoxaline, which has not been used so far for the synthesis of azomethine ylides, was triggered by the consideration that the initially formed spiroheterocyclic intermediate **7** can participate subsequently with phenylacetaldehyde through enamine reaction furnishing unusual dispiropyrrolidinyl-*N*-styrylpiperidone-indeno[1,2-*b*]quinoxaline heterocyclic hybrids (**8a**–**k)**. The phenylacetaldehyde **10** was generated in situ from the excess azomethine ylide **6** as shown in the mechanism (Scheme 4), which was further supported by our earlier report [19,20].

**Figure 2:**
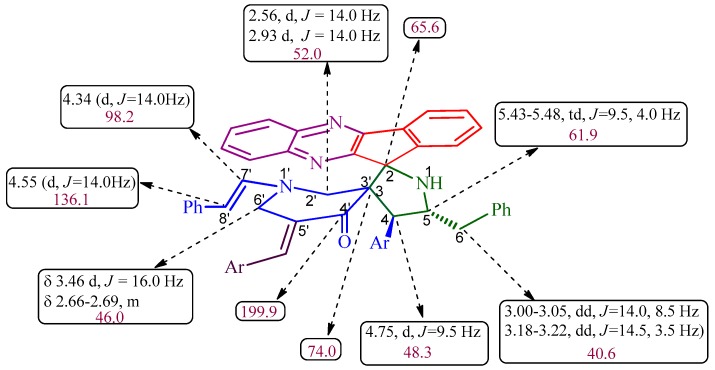
Selected ^1^H and ^13^C chemical shifts of **8j**.

**Figure 3:**
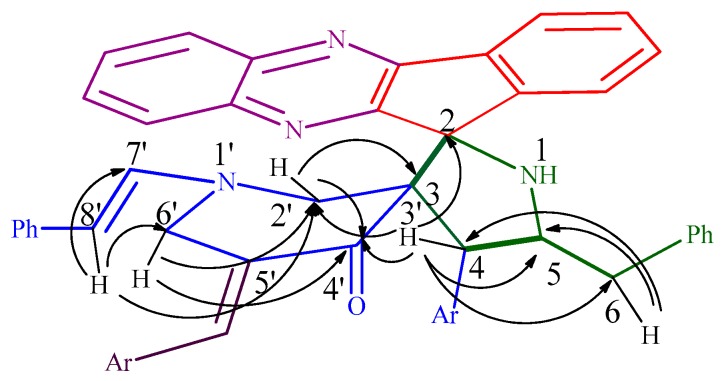
Selected HMBCs of **8j**.

The structure of dispiropyrrolidine tethered indeno[1,2-*b*]quinoxaline heterocyclic hybrids **8a**–**k** was elucidated with the help of ^1^H-, ^13^C- and 2D-NMR spectroscopic analysis. As a representative case, the structural assignment of **8j** is described below (Figure 2). In its ^1^H-NMR spectrum, the two doublet of doublets at δ 3.00–3.05 and 3.18–3.22 ppm are due to H-6 hydrogens, which shows ^1^H,^1^H-COSY correlation with the triplet of the doublet at δ 5.43–5.48 ppm, and, hence, it can be assigned to H-5 hydrogen. The H-5 hydrogen shows ^1^H-^1^H-COSY correlation (Figure 3) with H-4 which appears as a doublet at δ 4.75 ppm (*J* = 9.5 Hz). H-4 shows HMBCs (Heteronuclear Multi Bond Correlations) with C-2, C-5, C-6 and C-2′ at 65.6, 61.9, 40.6 and 52.0 ppm, respectively. H-2′ shows HMBCs with C-3 and C-4′ at 74.0 and 199.9 ppm and H-6 shows HMBCs with C-4 at 48.3 ppm. Further, the chemical shift of methine, methylene, and methoxy carbons has also been assigned by DEPT-135 analysis. H-7′ appeared as a doublet at δ 4.34 (*J*= 14.0 Hz) that shows ^1^H, ^1^H-COSY correlation with H-8′, which in turn shows HMBCs with C-7′ at δ 98.2 ppm. H-8′ shows HMBCs with C-7′ and C-6′ at 98.2 and 46.0 ppm respectively, was further confirmed by the styryl unit attached in the *N*-piperidinone moiety of cycloadduct. The chemical shifts of hydrogens and C,H-COSY correlations helped in the assignment of the hydrogen-bearing carbons. The structure deduced from NMR studies was in agreement with combustion microanalytical data and with the mass spectrum of **8j**. Further, the structure of the compound **8j** was unambiguously assigned based on our previous reports [19,20,23].

The above one-pot multicomponent reaction presumably proceeds through a domino sequence and a probable reaction mechanism is described in Scheme 4. Ionic liquid and [bmim]Br play a twin role as a solvent and catalyst, the electron deficient hydrogen atom of [bmim]Br could form the hydrogen bonds with carbonyl units of ninhydrin which would increase the electrophilicity of the carbonyl carbon, probably accelerating the reaction [24]. Presumably, the amine group of *o*-phenylenediamine **2** attacks the carbonyl group of ninhydrin **1** to furnish indeno[1,2-*b*]quinoxalin-11-one **3**. Compound **5** further reacted with the reactive azomethine ylide **6** via decarboxylative condensation. The azomethine ylide attacks regio-selectively the β-carbon of C=C bond to form **7**. Simultaneously, the ylide **6**′ was attacked by water molecule to furnish 2-phenylacetaldehyde **10** via **9**. Subsequently, the secondary amine of piperidone in the spirocycloadduct **7** was reacted with 2-phenylacetaldehyde **10** through enamine reaction to afford **8**.

### 2.2. Biological Evaluation

#### 2.2.1. Antibacterial Activities 

**Figure 4:**
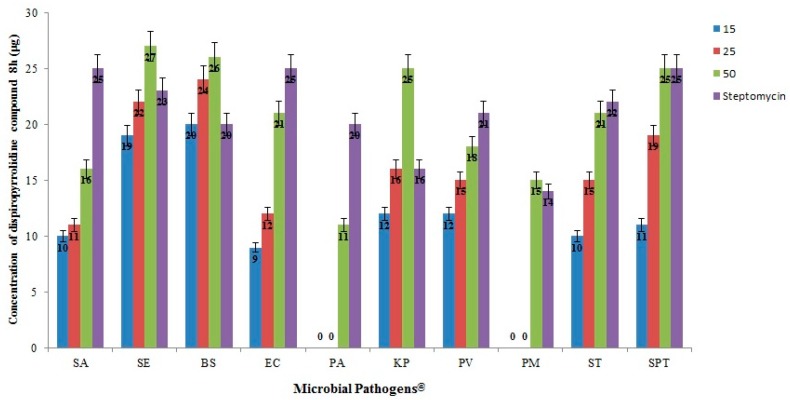
Antibacterial activity of dispiropyrrolidine compound **8h** against Gram-positive and negative bacterial pathogens. ^@^SA—*Staphylococcus aureus* MTCC 96, SE—*Staphylococcus epidermidis* MTCC 3615. BS—*Bacillus subtilis* MTCC 441, EC—*Escherichia coli* ATCC 25922, PS—*Pseudomonas aeruginosa* ATCC 27584, KP—*Klebsiella pneumoniae* MTCC 109. PV—*Proteus vulgaris* ATCC 8427, PM—*Proteus mirabilis* ATCC 25922, ST—*Salmonella typhi* ATCC 25922, SPT—*Salmonella paratyphi* MTCC 735.

The antibacterial activity of dispiropyrrolidine integrated indeno[1,2-*b*] quinoxaline heterocyclic hybrids **8a**–**k** was determined by agar well diffusion method [25] and the results were tabulated against three Gram-positive bacteria and seven Gram-negative bacteria (vide supplementary data, Tables S1 and S2). Among them, compounds **8a**, **8b**, **8h**, **8k**, and **8l** exhibited effective activities against all the three tested Gram-positive bacteria (vide supplementary data, Table S1). In particular, compound **8h** bearing *m*-methyl and **8k** with *p*-methoxy substituent showed a maximum of 26 and 25 mm zone against *Bacillus subtilis* MTCC 441, respectively. The minimum zone of inhibition was observed for **8d** and **8f** against *Staphylococcus aureus* MTCC 96 and *Staphylococcus epidermidis* MTCC 3615, respectively (vide supplementary data, Figure S8). Compounds **8a**–**k** were also tested against seven Gram-negative bacterial pathogens (vide supplementary data, Table S2). Therein four pathogens viz., *Proteus vulgaris* ATCC 8427, *Proteus mirabilis* ATCC 7002, *Salmonella typhi* ATCC 19430, and *Salmonella paratyphi* MTCC 735 effectively inhibited and observed maximum zone of inhibition compared with the other three pathogens namely, *Escherichia coli* ATCC 25922, *Pseudomonas aeruginosa* ATCC 27584, and *Klebsiella pneumoniae* MTCC 109. Particularly, the maximum number of dispiropyrrolidine compounds showed a smaller inhibition zone against *Pseudomonas aeruginosa* ATCC 27584. Compounds **8c**, **8f**, **8g**, **8h**, **8i**, and **8k** exhibited maximum zone of inhibition against *Salmonella paratyphi* MTCC 735. However, a minimum zone of inhibition was observed against *Pseudomonas aeruginosa* ATCC 27584 (vide supplementary, Figure S8). Overall, the dispiropyrrolidine compound **8h** exhibited competent antibacterial activity against both Gram-positive and negative bacterial pathogens (Figure 4). 

Minimum inhibition concentration (MIC) values of the synthesized compound **8h** along with streptomycin were presented in Table 2. Gram-positive bacteria, *S. epidermidis* MTCC 3615 and *B. subtilis* MTCC 441 displayed the MIC value of 31.25 µg/mL and *S. aureus* MTCC 96 MIC value was noted as 125.00 µg/mL. However, the MIC values of Gram-negative bacterial pathogens were ranged between 7.80 and 250.00 µg/mL. The minimum (7.80 µg/mL) and maximum (250.00 µg/mL) MIC value were observed against *K. pneumoniae* MTCC 109 and *P. aeruginosa* ATCC 27584, respectively. The MIC value of *S. typhi* ATCC 25922 and *P. vulgaris* ATCC 8427 were 15.60 µg/mL. Compound **8h** showed potent MIC values (7.80 µg/mL) against tested bacterial pathogens, particularly *K. pneumoniae* MTCC 109 and *S. epidermidis* MTCC 3615. MIC values of the compound **8h** were comparatively higher than the commercial antibiotic streptomycin against some tested bacterial pathogens (Table 2).

#### 2.2.2. Synergistic Activity 

The combination of compound **8h** with streptomycin and vancomycin acted synergistically against streptomycin, tetracycline, and vancomycin-resistant *E. coli* ATCC 25922. The FICI (Fractional Inhibitory Concentration Index) of each synergistic combination calculated from the results of the chequerboard assays is presented in Table 3. The MIC values of compound **8h** and streptomycin against *E. coli* ATCC 25922 were 62.50 and 10 µg/mL, respectively, whereas the MIC values of compound **8h** and streptomycin in synergistic combination against *E. coli* ATCC 25922 were 15.60 and 2.5 µg/mL, respectively. In addition, there was a significant decrease in MIC values in different combinations of compounds with vancomycin. The FICI of compound **8h** with various antibiotics such as streptomycin, tetracycline, and vancomycin were 0.5, 1.0, and 0.75, respectively (Table 3). The combinations of compound **8h** with antibiotics showed a remarkable effect in decreasing the MIC values. Furthermore, the synergistic effect showed that the presence of meta substituted aromatic ring could disturb the cytoplasmic membrane structure, thus causing loss of integrity and eventually the cell death. At sub-bactericidal concentrations of the dispiropyrrolidine compound **8h** might facilitate the entry of the antibiotic to the cell cytoplasm, thus assisting the passage of streptomycin and tetracycline, which have their site of action within the bacterial cell, and a lower antibiotic dose would be needed. In this aspect, both **8h** and the antibiotics exhibited a collective mechanism of actions; it would be attained by disrupting the bio-membrane and subsequently metabolic process such as protein synthesis, cell wall synthesis and DNA synthesis, based on the antibiotic used.

Hence, these results profoundly suggest that the combination of compound **8h** with streptomycin and vancomycin exhibited outstanding synergistic activity against *E. coli* ATCC 25922. Nevertheless, the combination of compound **8h** with tetracycline shows no synergistic activity against *E. coli*. In the future, the synergistic effect of compound **8h** with antibiotic against the multidrug-resistant bacteria may be useful for the treatment of infectious diseases. 

#### 2.2.3. Antifungal Activity

**Figure 5:**
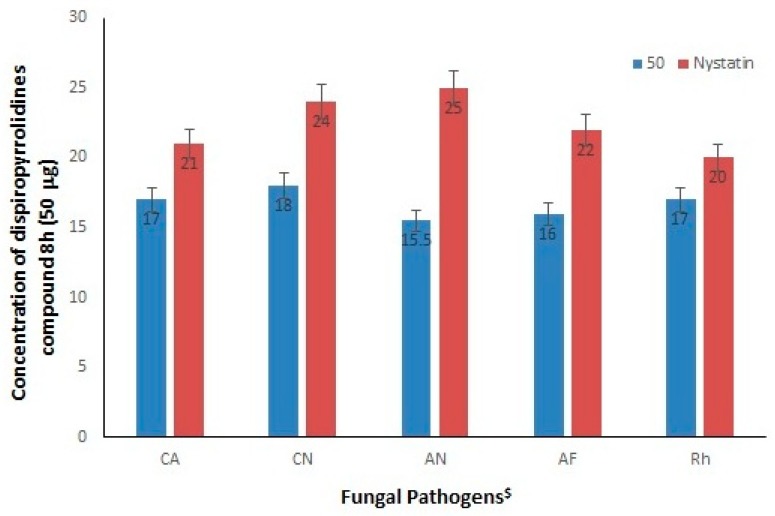
Antifungal activity of dispiropyrrolidine compound **8h** against fungal pathogens. ^$^CA*—Candida albicans* BL0142, CN—*Candida neoformans* BL1703, AF—*Aspergillus flavus* BL5064, AN—*Aspergillus niger* BL4217, Rh—*Rhizopus* sp. BL3389.

The antifungal activity profiles of the compounds **8a**–**k** and commercial antifungal drugs nystatin are shown in Supplementary Table S3. Among them, six compounds showed significant antifungal activity against tested clinical fungal strains. However, compounds **8e** bearing *p*-chloro, **8f** *o*,*m*-dichloro, **8h** *p*-methyl, and **8k** *p*-methoxy substituents on the aryl ring exhibited competent activity against *C. albicans* BL0142, *C. neoformans* BL1703, and *A. flavus* BL5064, whereas *A. niger* BL4217 and *Rhizopus* sp. BL3389 exhibited moderate to low activities (Figure 5). The inhibition zone range of commercial drug showed between 20.0 and 25.0 mm. 

### 2.3. Docking Simulation

**Figure 6:**
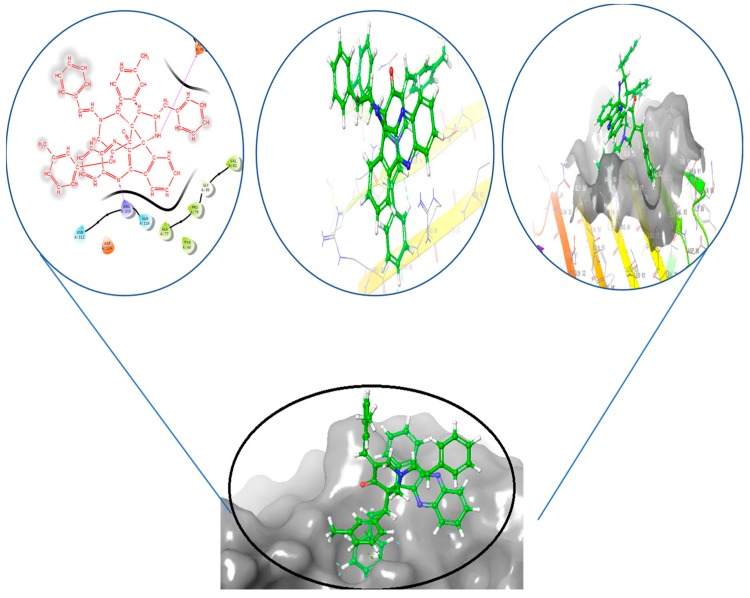
Molecular docking conformation of compound **8h** into the bacterial outer membrane protein (1IWN) protein binding pocket.

The most active dispiropyrrolidine, namely 8h was docked into the active site of experimentally known ligand obtained from RCSB (Research Collaboratory for Structural Bioinformatics). These strategies expected to disclose the binding affinity of this antimicrobial agent to their respective receptor and their relative orientation inside the receptor and in contact with active site residues. Molecular interaction protocols set up based on Schrödinger (2018-2) program [26,27]. Docking simulation scores were analyzed and detailed in Table 4. Docking studies against (1IWN) Outer Membrane Lipoprotein Receptor [28] revealed that compound **8h** interacted with the side chain hydrogen bond (ASP96, ARG115) on amine group and benzyl nitrogen atom with electrically charged amino acids. The binding site residues of ASN 112, ASP 124, GLN116, ALA77, TYR60, PRO79, GLY80, VAL82, ALA97, ASP95 were shown to be hydrophobic interactions to hold the molecular orientation to express the bimolecular activity (Figure 6). The docking result of the compound showed the binding affinity with GLIDE score of −4.376 with a binding energy of −39.576 (Kcal/mol). The result revealed that compound **8h** interacted strongly with biochemical bonding interaction and it helps to have good pharmacological inhibitory activity against microbial pathogens. To summarize, the synthetic compound had a good binding affinity with the molecular interaction energy score, and thereby the compound can act as a superlative ligand with inhibitory activity against gram-negative bacterial pathogens. 

## 3. Material and Methods

### General Procedure for Synthesis of Dispiropyrrolidine Heterocyclic hybrids, ***8a**–**k***

A mixture of 3,5-diarylidenepiperidin-4-ones (0.727 mmol), ninhydrin (0.727 mmol), *o*-phenylenediamine (0.727 mmol) and l-phenylalanine (1.45 mmol) in 200 mg of [bmim]Br and reaction mixture was heated for 1 h. After completion of the reaction as evident by TLC analysis, the crude product was purified by column chromatography. 

*5-Benzyl-spiro-[2.11′]-indeno-[1,2-b]quinoxaline-spiro-[3.3′]benzylidine-N-strylpiperidone-4-phenyl-pyrrolidine* (**8a**). Yield 51%; Yellow solid: m.p. 145–147 °C; ^1^H-NMR (CDCl_3_, 400 MHz): δ (ppm) 2.02 (d, *J* = 14.0 Hz, 1H), 2.33 (d, *J* = 14.0 Hz, 1H), 2.88–2.90 (m, 1H), 3.38 (d, *J* = 16.0 Hz, 1H), 3.60 (d, *J* = 14.0 Hz, 1H), 3.69 (d, *J* = 14.0 Hz, 1H), 4.54 (d, *J* = 14.0 Hz, 1H), 4.67 (d, *J* = 10.0 Hz, 1H), 4.84 (d, *J* = 14.0 Hz, 1H), 5.33–5.39 (td, *J* = 10.0, 3.6 Hz, 1H), 6.07–6.09 (m, 2H), 6.63–8.30 (m, 27H, ArH); ^13^C-NMR (CDCl_3_, 100 MHz): δ (ppm) 39.4, 46.8, 53.2, 53.7, 61.5, 66.8, 72.9, 100.6, 121.8, 123.8, 126.3, 126.3, 127.2, 127.8, 128.3, 128.7, 128.8, 128.9, 129.1, 129.2, 129.4, 129.6, 129.8, 130.3, 130.9, 131.0, 131.6, 132.1, 134.5, 136.3, 136.7, 137.8, 138.7, 139.7, 140.8, 141.8, 146.9, 154.1, 163.7, 197.8. LC/MS(ESI): **m*/*z** = 712 (M^+^); Anal. Calcd for C_50_H_40_N_4_O: C, 84.24; H, 5.66; N, 7.86; Found C, 84.35; H, 5.78; N, 7.95%.

*5-Benzyl-spiro-[2.11′]-indeno-[1,2-b]quinoxaline-spiro-[3.3′]2-bromo-benzylidine-N-strylpiperidone-4-[2-bromophenyl]-pyrrolidine* (**8b**). Yield 52%; Pale Yellow solid: m.p. 171–173 °C; δ (ppm) ^1^H-NMR (CDCl_3_, 400 MHz): δ (ppm) 2.26 (d, *J* = 14.0 Hz, 1H), 2.65–2.70 (d, *J* = 14.0, 7.2 Hz, 1H), 2.81–2.86 (dd, *J* = 14.0, 8.0 Hz, 1H), 2.94–2.98 (dd, *J* = 14.0, 3.6 Hz, 1H), 3.29 (d, *J* = 16.0 Hz, 1H), 3.55 (d, *J* = 14.0 Hz, 1H), 4.47 (d, *J* = 14.0 Hz, 1H), 4.54 (d, *J* = 10.0 Hz, 1H), 4.76 (d, *J* = 14.0 Hz, 1H), 5.08–5.11 (td, *J* = 8.0, 3.6 Hz, 1H), 6.23 (m, 2H), 6.79–8.20 (m, 25H, ArH); ^13^C-NMR (CDCl_3_, 100MHz): δ (ppm) 39.6, 46.8, 48.5, 52.1, 62.1, 66.2, 74.2, 97.9, 120.9, 121.2, 122.3, 122.8, 123.1, 123.8, 124.0, 125.9, 126.3, 126.5, 127.2, 127.9, 128.3, 128.4, 128.5, 129.2, 129.3, 129.4, 130.4, 130.8, 130.9, 131.2, 134.8, 136.5, 137.8, 138.4, 139.8, 140.8, 141.9, 145.6, 154.0, 158.1, 158.5, 163.7, 199.9. LC/MS(ESI): *m*/*z* = 870 (M^+^); Anal. Calcd for C_50_H_38_Br_2_N_4_O: C, 68.97; H, 4.40; N, 6.43; Found C, 69.08; H, 4.52; N, 6.51%.

*5-Benzyl-spiro-[2.11′]-indeno-[1,2-b]quinoxaline-spiro-[3.3′]-4-bromo-benzylidine-N-strylpiperidone-4-[4-bromophenyl]-pyrrolidine* (**8c**). Yield 55%; Pale yellow solid: m.p. 165–167 °C; ^1^H-NMR (CDCl_3_, 400 MHz): δ (ppm) 2.33 (d, *J* = 14.0 Hz, 1H), 2.88–2.92 (dd, *J* = 14.0, 7.2 Hz, 1H), 3.11–3.16 (m, 2H), 3.34 (d, *J* = 16.0 Hz, 1H), 3.61 (d, *J* = 14.0 Hz, 1H), 4.53 (d, *J* = 14.0 Hz, 1H), 4.61 (d, *J* = 10.0 Hz, 1H), 4.82 (d, *J* = 14.0 Hz, 1H), 5.15–5.19 (td, *J* = 9.0, 3.6 Hz, 1H), 6.10–6.12 (m, 2H), 6.86–8.27 (m, 25H, ArH); ^13^C-NMR (CDCl_3_, 100 MHz): δ (ppm) 40.1, 46.2, 48.2, 52.1, 61.8, 65.6, 74.2, 98.2, 122.1, 123.7, 123.9, 126.4, 127.3, 127.4, 128.5, 128.6, 128.7, 128.8, 129.2, 129.3, 129.4, 129.5, 130.3, 130.9, 131.3, 132.5, 134.5, 136.4, 137.9, 138.4, 139.2, 140.5, 140.8, 141.7, 154.7, 158.5, 160.2, 165.2, 199.1. LC/MS(ESI): *m*/*z* = 870 (M^+^); Anal. Calcd for C_50_H_38_Br_2_N_4_O: C, 68.97; H, 4.40; N, 6.43; Found C, 69.05; H, 4.53; N, 6.52%.

*5-Benzyl-spiro-[2.11′]-indeno-[1,2-b]quinoxaline-spiro-[3.3′]-2-chloro-benzylidine-N-strylpiperidone-4-[2-chlorophenyl]-pyrrolidine* (**8d**). Yield 50%; Yellow solid: m.p. 149–151 °C; ^1^H-NMR (CDCl_3_, 400 MHz): δ (ppm) 2.50 (d, *J* = 14.0 Hz, 1H), 2.62–2.66 (m, 1H), 2.85 (d, *J* = 14.0 Hz, 1H), 3.05–3.06 (m, 2H), 3.33 (d, *J* = 16.0 Hz, 1H), 4.25 (d, *J* = 14.0 Hz, 1H), 4.49 (d, *J* = 14.0 Hz, 1H), 4.87 (d, *J* = 9.5 Hz, 1H), 5.23–5.30 (m, 1H), 6.13–6.14 (m, 2H), 6.74–8.53 (m, 25H, ArH); ^13^C-NMR (CDCl_3_, 100MHz): δ (ppm) 40.5, 45.7, 51.6, 52.3, 64.2, 65.9, 74.4, 98.9, 122.3, 123.7, 126.2, 126.4, 126.6, 127.0, 128.1, 128.3, 128.9, 129.2, 129.4, 129.6, 129.8, 130.7, 131.3, 132.3, 133.1, 134.9, 135.8, 136.1, 136.2, 136.4, 136.5, 137.7, 138.0, 138.5, 140.5, 141.8, 144.7, 145.8, 154.7, 158.5, 158.6, 162.8, 199.1. LC/MS(ESI): *m*/*z* = 781 (M^+^); Anal. Calcd for C_50_H_38_Cl_2_N_4_O: C, 76.82; H, 4.90; N, 7.17; Found C, 76.94; H, 4.99; N, 7.26%.

*5-Benzyl-spiro-[2.11′]-indeno-[1,2-b]quinoxaline-spiro-[3.3′]-2,4-dichloro-benzylidine-N-strylpiperidone-4-[2,4-dichlrophenyl]-pyrrolidine* (**8e**). Yield 48%; Yellow solid: m.p. 180–182 °C; ^1^H-NMR (CDCl_3_, 400 MHz): δ (ppm) 2.37 (m, 1H), 2.86–2.92 (dd, *J* = 14.0, 8.0 Hz, 1H), 3.16–3.22 (m, 2H), 3.40 (d, *J* = 16.0 Hz, 1H), 3.66 (d, *J* = 14.0 Hz, 1H), 4.54 (d, *J* = 14.0 Hz, 1H), 4.63 (d, *J* = 10.0 Hz, 1H), 4.85 (d, *J* = 14.0 Hz, 1H), 5.20–5.25 (td, *J* = 8.0, 3.6 Hz, 1H), 6.11–6.12(m, 2H), 6.65–8.51 (m, 25H, ArH); ^13^C-NMR (CDCl_3_, 100 MHz): δ (ppm) 40.3, 46.8, 50.4, 50.9, 63.7, 66.4, 74.5, 98.5, 122.1, 123.8, 126.5, 127.2, 127.4, 128.1, 128.5, 128.9, 129.3, 129.4, 129.5, 129.8, 129.9, 130.7, 131.6, 132.5, 132.6, 134.4, 135.4, 136.1, 136.5, 136.6, 136.9, 137.8, 138.2, 140.2, 141.8, 145.7, 154.7, 158.3, 158.9, 163.8, 196.5. LC/MS(ESI): *m*/*z* = 850 (M^+^); Anal. Calcd for C_50_H_36_Cl_4_N_4_O: C, 70.60; H, 4.27; N, 6.59; Found C, 70.73; H, 4.38; N, 6.70%.

*5-Benzyl-spiro-[2.11′]-indeno-[1,2-b]quinoxaline-spiro-[3.3′]-4-chloro-benzylidine-N-strylpiperidone-4-[4-chlorophenyl]-pyrrolidine* (**8f**). Yield 61%; Yellow solid: m.p. 151–153 °C; ^1^H-NMR (CDCl_3_, 400MHz): δ (ppm) 2.26 (d, *J* = 14.0 Hz, 1H), 2.77–2.86 (m, 1H), 3.02–3.11(m, 2H), 3.28 (d, *J* = 16.0 Hz, 1H), 3.56 (d, *J* = 14.0 Hz, 1H), 4.47 (d, *J* = 14.0 Hz, 1H), 4.55 (d, *J* = 10.0 Hz, 1H), 4.76 (d, *J* = 14.0 Hz, 1H), 5.08–5.14 (td, *J* = 10.0, 3.6 Hz, 1H), 6.02–6.04 (m, 2H), 6.79–8.20 (m, 25H, ArH); ^13^C-NMR (CDCl_3_, 100MHz): δ (ppm) 39.6, 46.8, 52.6, 52.9, 62.2, 66.6, 72.6, 98.7, 123.8, 124.0, 126.4, 127.2, 127.9, 128.4, 128.8, 128.9, 129.1, 129.2, 129.3, 129.5, 130.7, 130.9, 131.1, 131.3, 132.8, 133.1, 134.7, 135.4, 135.9, 137.3, 137.5, 138.4, 138.5, 141.9, 146.5, 153.8, 158.6, 163.2, 199.7. LC/MS(ESI): *m*/*z* = 781 (M^+^); Anal. Calcd for C_50_H_38_Cl_2_N_4_O: C, 76.82; H, 4.90; N, 7.17; Found C, 76.93; H, 4.97; N, 7.28%.

*5-Benzyl-spiro-[2.11′]-indeno-[1,2-b]quinoxaline-spiro-[3.3′]-3-methyl-benzylidine-N-strylpiperidone-4-[2-methylphenyl]-pyrrolidine* (**8g**). Yield 54%; Yellow solid: m.p. 135–137 °C; ^1^H-NMR (CDCl_3_, 400MHz): δ (ppm) 2.19 (s, 3H), 2.31 (s, 3H), 2.37 (d, *J* = 14.0 Hz, 1H), 2.78–2.82 (m, 1H), 2.96–3.01 (dd, *J* = 14.0, 8.0 Hz, 1H), 3.12–3.16 (dd, *J* = 14.0, 3.5 Hz, 1H), 3.30–3.37 (m, 2H), 4.37 (d, *J* = 14.0 Hz, 1H), 4.65 (d, *J* = 14.0 Hz, 1H), 4.79 (d, *J* = 9.5 Hz, 1H), 5.35–5.41 (td, *J* = 9.5, 4.0 Hz, 1H), 6.12–6.14 (m, 2H), 6.67–8.53 (m, 25H, ArH); ^13^C-NMR (CDCl_3_, 100 MHz): δ (ppm) 19.9, 21.0, 40.0, 45.8, 50.2, 53.0, 64.4, 65.8, 76.7, 99.3, 122.1, 123.6, 125.6, 126.1, 126.2, 126.8, 127.3, 127.9, 128.2, 128.3, 128.9, 129.1, 129.3, 129.4, 129.5, 130.3, 130.5, 130.9, 131.1, 133.6, 135.9, 136.3, 137.6, 138.0, 138.3, 138.6, 138.9, 140.6, 141.7, 145.5, 163.7, 199.2. LC/MS(ESI): *m*/*z* = 740 (M^+^); Anal. Calcd for C_52_H_44_N_4_O: C, 84.29; H, 5.99; N, 7.56; Found C, 84.41; H, 6.11; N, 7.68%.

*5-Benzyl-spiro-[2.11′]-indeno-[1,2-b]quinoxaline-spiro-[3.3′]-3-methyl-benzylidine-N-strylpiperidone-4-[3-methylphenyl]-pyrrolidine* (**8h**). Yield 58%; Pale Yellow solid: m.p. 139–141 °C; ^1^H-NMR (CDCl_3_, 500MHz): δ (ppm) 2.24 (s, 3H), 2.31 (s, 3H), 2.35 (d, *J* = 14.0 Hz, 1H), 2.88–2.92 (dd, *J* = 14.0, 8.0 Hz, 1H), 3.15–3.22 (m, 2H), 3.40 (dd, *J* = 16.0 Hz, 1H), 3.68 (d, *J* = 14.0 Hz, 1H), 4.53 (d, *J* = 14.0 Hz, 1H), 4.63 (d, *J* = 10.0 Hz, 1H), 4.85 (d, *J* = 14.0 Hz, 1H), 5.21–5.25 (td, *J* = 8.0, 3.5 Hz, 1H), 6.08–6.09 (m, 2H), 6.79–8.30 (m, 25H, ArH); ^13^C-NMR (CDCl_3_, 125 MHz): δ (ppm) 21.5, 21.8, 39.6, 47.0, 53.1, 53.4, 62.1, 66.8, 72.9, 100.9, 122.0, 123.8, 123.9, 124.9, 126.3, 127.0, 127.1, 127.9, 128.0, 128.4, 128.6, 128.9, 129.0, 129.3, 129.4, 129.5, 129.8, 129.9, 130.1, 130.3, 131.0, 131.3, 134.4, 136.4, 137.3, 137.6, 137.9, 138.1, 138.2, 138.9, 139.4, 141.0, 141.9, 146.9, 154.2, 165.3, 198.0. LC/MS(ESI): *m*/*z* = 740 (M^+^); Anal. Calcd for C_52_H_44_N_4_O: C, 84.29; H, 5.99; N, 7.56; Found C, 84.40; H, 6.11; N, 7.64%.

*5-Benzyl-spiro-[2.11′]-indeno-[1,2-b]quinoxaline-spiro-[3.3′]-4-methyl-benzylidine-N-strylpiperidone-4-[4-methylphenyl]-pyrrolidine* (**8i**). Yield 60%; Pale Yellow solid: m.p. 142–144 °C; ^1^H-NMR (CDCl_3_, 400 MHz): δ (ppm) 2.18 (s, 3H), 2.29 (s, 3H), 2.36 (d, *J* = 14.0 Hz, 1H), 2.78–2.82 (m, 1H), 2.94–3.00 (dd, *J* = 14.0, 8.0 Hz, 1H), 3.10–3.14 (dd, *J* = 14.0, 3.5 Hz, 1H), 3.29–3.36 (m, 2H), 4.35 (d, *J* = 14.0 Hz, 1H), 4.64 (d, *J* = 14.0 Hz, 1H), 4.78 (d, *J* = 9.6 Hz, 1H), 5.20–5.25 (td, *J* = 9.6, 3.6 Hz, 1H), 6.11–6.12(m, 2H), 6.65–8.51 (m, 25H, ArH); ^13^C-NMR (CDCl_3_, 400 MHz): δ (ppm) 21.1, 21.4, 39.4, 46.9, 52.9, 53.1, 61.9, 66.7, 72.7, 100.7, 121.9, 123.7, 126.2, 127.2, 127.8, 128.4, 128.8, 128.9, 129.2, 129.3, 129.4, 129.5, 129.8, 130.3, 131.1, 131.7, 132.1, 134.5, 136.3, 136.7, 137.2, 137.8, 138.8, 139.2, 139.7, 140.9, 141.8, 146.8, 154.0, 163.9, 197.9. LC/MS(ESI): *m*/*z* = 740 (M^+^); Anal. Calcd for C_52_H_44_N_4_O: C, 84.29; H, 5.99; N, 7.56; Found C, 84.40; H, 6.09; N, 7.64%.

*5-Benzyl-spiro-[2.11′]-indeno-[1,2-b]quinoxaline-spiro-[3.3′]-2-methoxybenzylidine-N-strylpiperidone-4-[2-methoxyphenyl]-pyrrolidine* (**8j**). Yield 64%; Pale yellow solid: m.p. 191–193 °C; ^1^H-NMR (CDCl_3_, 500 MHz): δ (ppm) 2.56 (d, *J* = 14.0 Hz, 1H), 2.68 (m, 1H), 2.93 (d, *J* = 14.0 Hz, 1H), 3.00–3.05 (dd, *J* = 14.0, 8.5 Hz, 1H), 3.18–3.22 (dd, *J* = 14.5, 3.5 Hz, 1H), 3.46 (d, *J* = 16.0 Hz, 1H), 3.76 (s, 3H), 3.81 (s, 3H), 4.34 (d, *J* = 14.0 Hz, 1H), 4.55 (d, *J* = 14.0 Hz, 1H), 4.75 (d, *J* = 9.5 Hz, 1H), 5.43–5.48 (td, *J* = 9.5, 4.0 Hz, 1H), 6.22–6.24 (m, 2H), 6.80–8.18 (m, 25H, ArH); ^13^C-NMR (CDCl_3_, 125 MHz): δ (ppm) 40.6, 46.0, 48.3, 52.0, 55.0, 55.4, 61.9, 65.6, 74.0, 98.2, 109.8, 110.7, 120.2, 120.6, 122.1, 123.3, 123.6, 123.9, 126.2, 126.8, 127.3, 127.8, 128.0, 128.4, 128.7, 128.8, 129.2, 129.5, 130.2, 130.8, 131.1, 131.2, 134.4, 136.1, 137.8, 138.5, 139.6, 140.7, 141.8, 145.5, 154.1, 158.3, 158.4, 163.6, 199.9. LC/MS(ESI): *m*/*z* = 772 (M^+^); Anal. Calcd for C_52_H_44_N_4_O_3_: C, 80.80; H, 5.74; N, 7.25; Found C, 80.90; H, 5.87; N, 7.37%.

*5-Benzyl-spiro-[2.11′]-indeno-[1,2-b]quinoxaline-spiro-[3.3′]-4-methoxy-benzylidine-N-strylpiperidone-4-[4-methoxyphenyl]-pyrrolidine* (**8k**). Yield 56%; Pale yellow solid: m.p. 186–187 °C; ^1^H-NMR (CDCl_3_, 500 MHz): δ (ppm) 2.38 (d, *J* = 14.0 Hz, 1H), 2.88–2.93 (dd, *J* = 14.0, 8.0 Hz, 1H), 3.16–3.19 (m, 2H), 3.41 (d, *J* = 16.0 Hz, 1H), 3.64 (d, *J* = 14.0 Hz, 1H), 3.81 (s, 3H), 3.80 (s, 3H), 4.56 (d, *J* = 14.0 Hz, 1H), 4.63 (d, *J* = 10.0 Hz, 1H), 4.86 (d, *J* = 14.0 Hz, 1H), 5.17–5.20 (td, *J* = 8.0, 3.5 Hz, 1H), 6.10–6.12 (m, 2H), 6.82–8.30 (m, 25H, ArH); ^13^C-NMR (CDCl_3_, 125 MHz): δ (ppm) 39.6, 47.1, 52.7, 53.2, 55.3, 55.4, 62.3, 66.6, 72.9, 100.7, 114.1, 121.9, 123.8, 126.3, 127.3, 128.0, 128.3, 128.5, 128.9, 129.0, 129.4, 129.5, 129.7, 131.2, 132.4, 136.4, 137.3, 138.0, 138.9, 139.0, 141.0, 141.9, 146.9, 154.2, 158.8, 160.5, 165.4, 197.9 LC/MS(ESI): *m*/*z* = 772 (M^+^); Anal. Calcd for C_52_H_44_N_4_O_3_: C, 80.80; H, 5.74; N, 7.25; Found C, 80.91; H, 5.84; N, 7.35%.

## 4. Conclusions

In conclusion, we have developed an environmentally benign one-pot four component domino protocol for the synthesis of dispiropyrrolidine integrated indeno[1,2-*b*] quinoxaline heterocyclic hybrids in moderate to good yields. This domino process involved 1,3-dipolar cycloaddition and concomitant enamine reaction. The compounds thus synthesized were examined for their antimicrobial efficacy against ten bacterial and five fungal pathogens. All the heterocyclic hybrids showed effective activity, and, particularly, compound **8h** exhibited more significant activity against the tested microbial pathogens. The synergistic effect revealed that the combinations of compound **8h** with streptomycin and vancomycin exhibited outstanding synergistic activity against *E. coli* ATCC 25922. This synergistic effect might be due to the joint action of **8h** and respective antibiotics. Therefore, these combinations are acceptable candidates for testing with an animal model to enhance their activities and also restore the currently unused drugs due to the resistance phenomenon. The binding energy of −39.576 (Kcal/mol) displayed that compound **8h** interacted strongly with biochemical bonding interaction and helps to have good pharmacological inhibitory activity against microbial pathogens.

## Figures and Tables

**Table 1:** Solvent optimization of cycloadduct **8j**.

Entry	Solvents	Time (h)	Yield (%)
1	methanol	3	49
2	ethanol	3	45
3	acetonitrile	3	42
4	1,4-dioxane	3	40
5	1,4-dioxane: Methanol (1:1*V*/*V*)	3	46
6	[bmim]Br	1	64

**Table 2:** Minimum inhibitory concentration of dispiropyrrolidines compound **8h** and streptomycin.

Bacterial Pathogens	MIC µg/mL	
	Compound 8h	Streptomycin
**Gram-positive bacterial pathogens**		
*Staphylococcus aureus* MTCC 96	125.00	10.0
*Staphylococcus epidermidis* MTCC 3615	31.25	5.0
*Bacillus subtilis* MTCC 441	31.25	5.0
**Gram-negative bacterial pathogens**		
*Escherichia coli* ATCC 25922	62.50	5.0
*Pseudomonas aeruginosa* ATCC 27584	250.00	10.0
*Klebsiella pneumoniae* MTCC 109	15.60	5.0
*Proteus vulgaris* ATCC 8427	62.50	10.0
*Proteus mirabilis* ATCC 7002	125.00	10.0
*Salmonella typhi* ATCC 19430	15.60	5.0
*Salmonella paratyphi* MTCC 735	31.25	5.0

**Table 3:** FIC and FICI of streptomycin, tetracycline, and vancomycin combined with the compound **8h** of against *Escherichia coli* ATCC 25922.

Compound and Antibiotics Combinations (µg/mL)	MIC^a^	MIC^b^	FIC	FICI
Compound **8h**-Streptomycin				0.5
Compound **8h**	62.50	15.60	0.25	
Streptomycin	10.0	2.5	0.25	
Compound **8h**-Tetracycline				1.0
Compound **8h**	62.50	31.25	0.25	
Tetracycline	15.0	15.0	0.5	
Compound **8h**-Vancomycin				0.75
Compound **8h**	62.50	31.25	0.5	
Vancomycin	30.0	7.5	0.25	

MIC^a^—MIC of sample alone; MIC^b^—MIC of effect combinations.

**Table 4:** Molecular docking data of compound **8h** with bacteria outer membrane (1IWN) protein receptor.

	Molecular Docking			
Compound	Glide Score (Kcal/mol)	Emodel Score	Glide Energy	XP Hydrogen Bond
**8h**	−4.376	−48.79	−39.576	2 (ASP96, ARG115)

## Supplementary Materials

The supplementary materials are available.
