# Supplementary material for: Domino Multicomponent Approach for the Synthesis of Functionalized Spiro-Indeno[1,2-b]quinoxaline Heterocyclic Hybrids and Their Antimicrobial Activity, Synergistic Effect and Molecular Docking Simulation
Source: Molecules. 2019 May 22;24(10):1962. doi: 10.3390/molecules24101962 (PMC6572414; doi:10.3390/molecules24101962)
Supplement: Supplementary file 1 [file molecules-24-01962-s001.pdf]

Domino multicomponent approach for the synthesis of functionalized spiro-indeno[1,2-*b*]quinoxaline heterocyclic hybrids and their antimicrobial activity, synergistic effect and molecular docking simulation

Abdulrahman I. Almansour[1], Natarajan Arumugam[1,*], Raju Suresh Kumar[1], Dhaifallah M. Al-thamili[1], Govindasami Periyasami[1], Karuppiah Ponmurugan[2], Naif Abdullah Al-Dhabi[2], Karthikeyan Perumal[3] and Dhanaraj Premnath[4]

[1] Department of Chemistry, College of Science, King Saud University, P.O Box 2455, Riyadh 11451, Saudi Arabia; almansor@ksu.edu.sa (A.I.A); sraju@ksu.edu.sa; daife54321@hotmail.com (D.M.A.); pkandhan@ksu.edu.sa (G.P)
[2] Department of Botany and Microbiology, College of Science, King Saud University, Riyadh 11451, Saudi Arabia; pkaruppiah@ksu.edu.sa (K.P.M); naldhabi@ksu.edu.sa
[3] Department of Chemistry and Biochemistry, The Ohio State University, 151 W. Woodruff Ave, Columbus, Ohio 43210, USA; pkarthikjaya@gmail.com (K.P)
[4] Department of Bioscience and Technology, Karunya Institute of Technology and Science, Branch of Bioinformatics, School of Agriculture and Biosciences, Karunya Nagar, coimbatore-641114, India; prems.bioinfo@gmail.com
* Correspondence: anatarajan@ksu.edu.sa; Tel.: +966-114675907

S.No	List of Figures	Pages No.
1	^{1}H NMR spectrum of **8j**	S3
2	^{13}C NMR spectrum of **8j**	S4
3	DEPT-135 spectrum of **8j**	S5
4	^{1}H, ^{1}H-COSY spectrum of **8j**	S6
5	HMQC spectrum of **8j**	S7
6	HMBC spectrum of **8j**	S8
7	Mass spectrum of **8j**	S9

Experimental

2.1. General Methods

Melting points were measured using open capillary tubes and are uncorrected. ^{1}H and ^{13}C NMR spectrawere recorded on a Varian Mercury JEOL-400 NMR and 500 NMR spectrometers in $CDCl_3$ using TMS as internal standard. Chemical shifts are given in parts per million (δ-scale) and coupling constants are given in hertz. Elemental analyses were performed, n a Perkin Elmer 2400 Series II Elemental CHNS analyser. Mass spectra were recorded on a Quattro Premier™ instrument (Micromass, Milford, USA) equipped with an electrospray ionization source (Zespray) coupled with an Acquity® UPLC system.

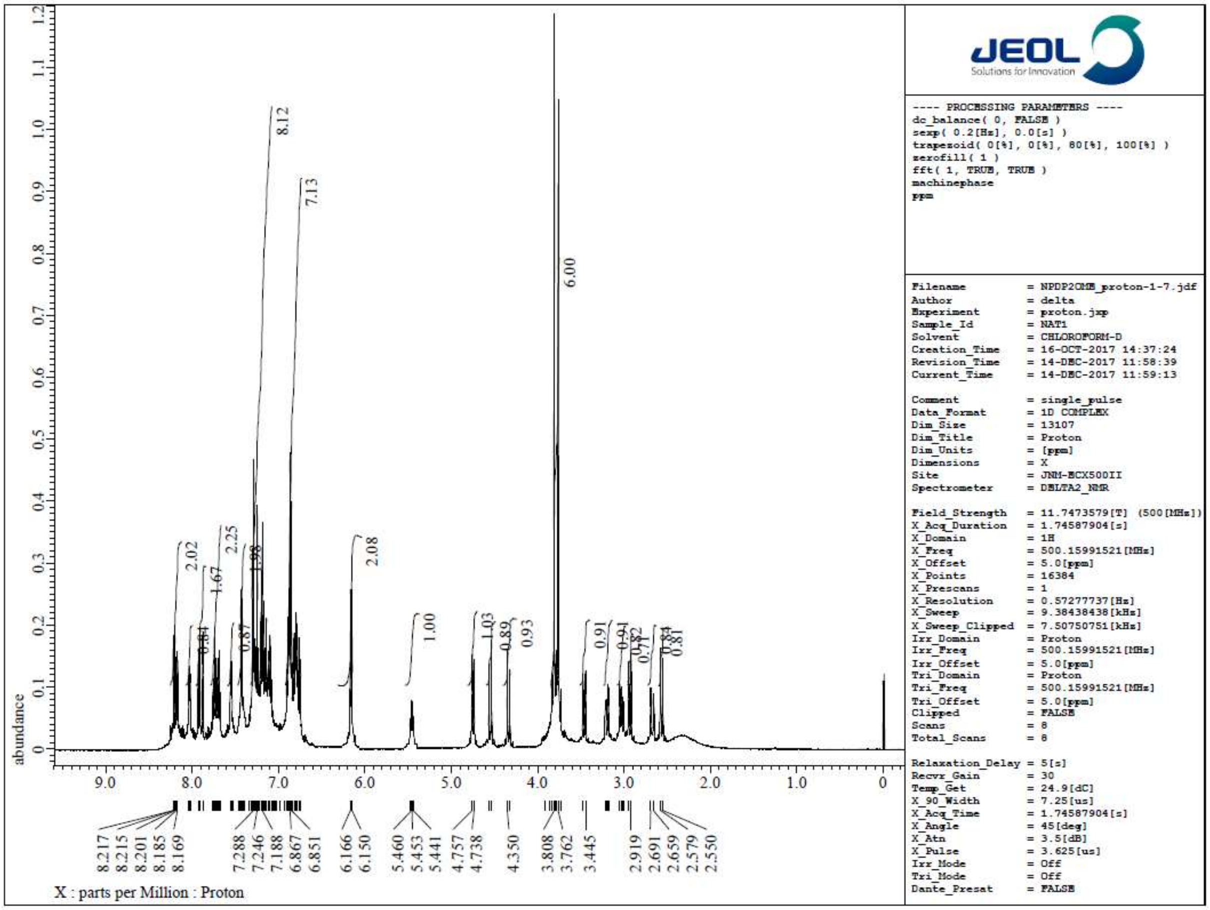

Figure S1. ^{1}H NMR spectrum of **8j**

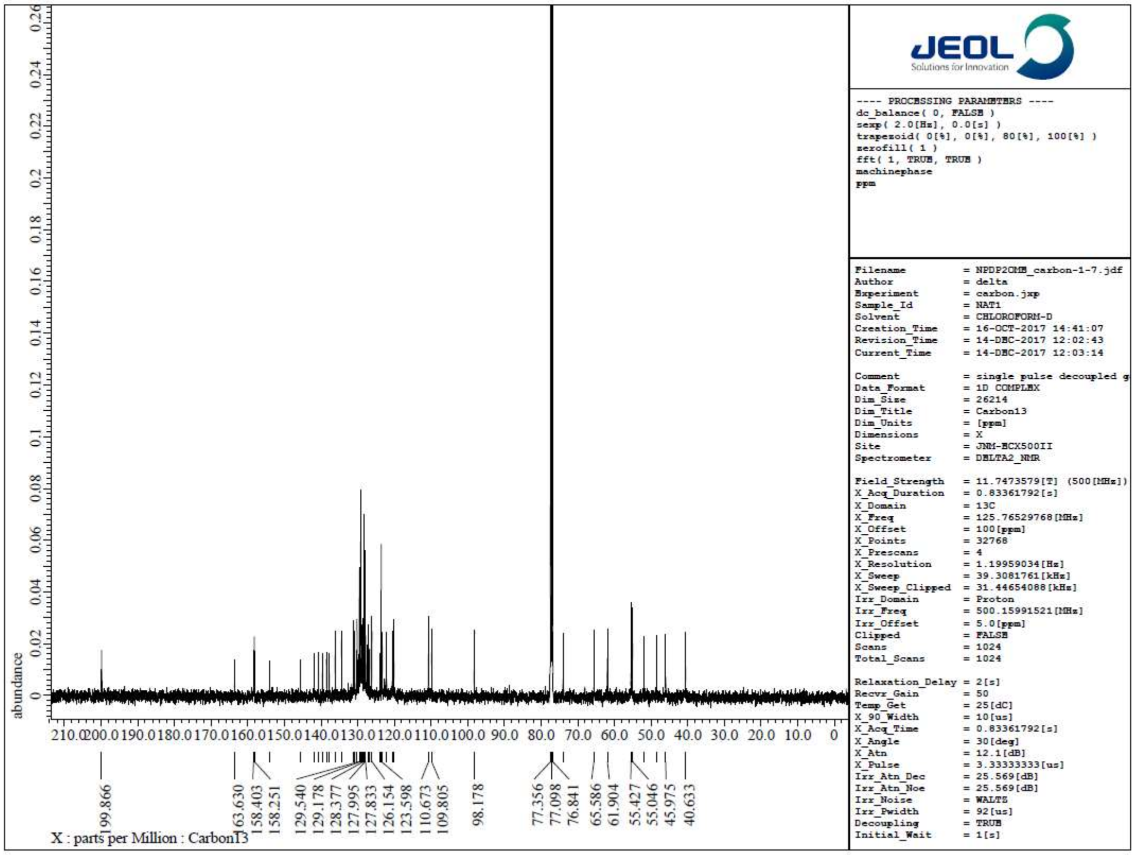

Figure S2. ^{13}C NMR spectrum of **8j**

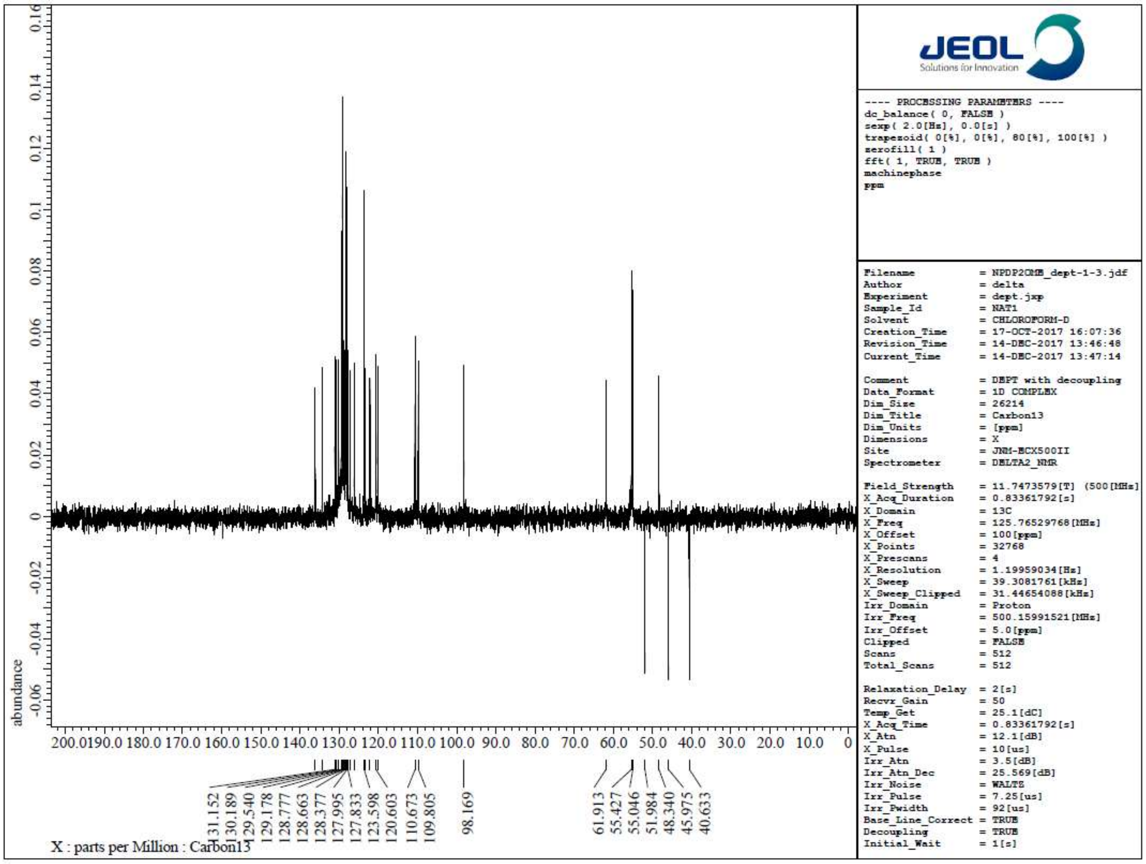

Figure S3. DEPT-135 spectrum of **8j**

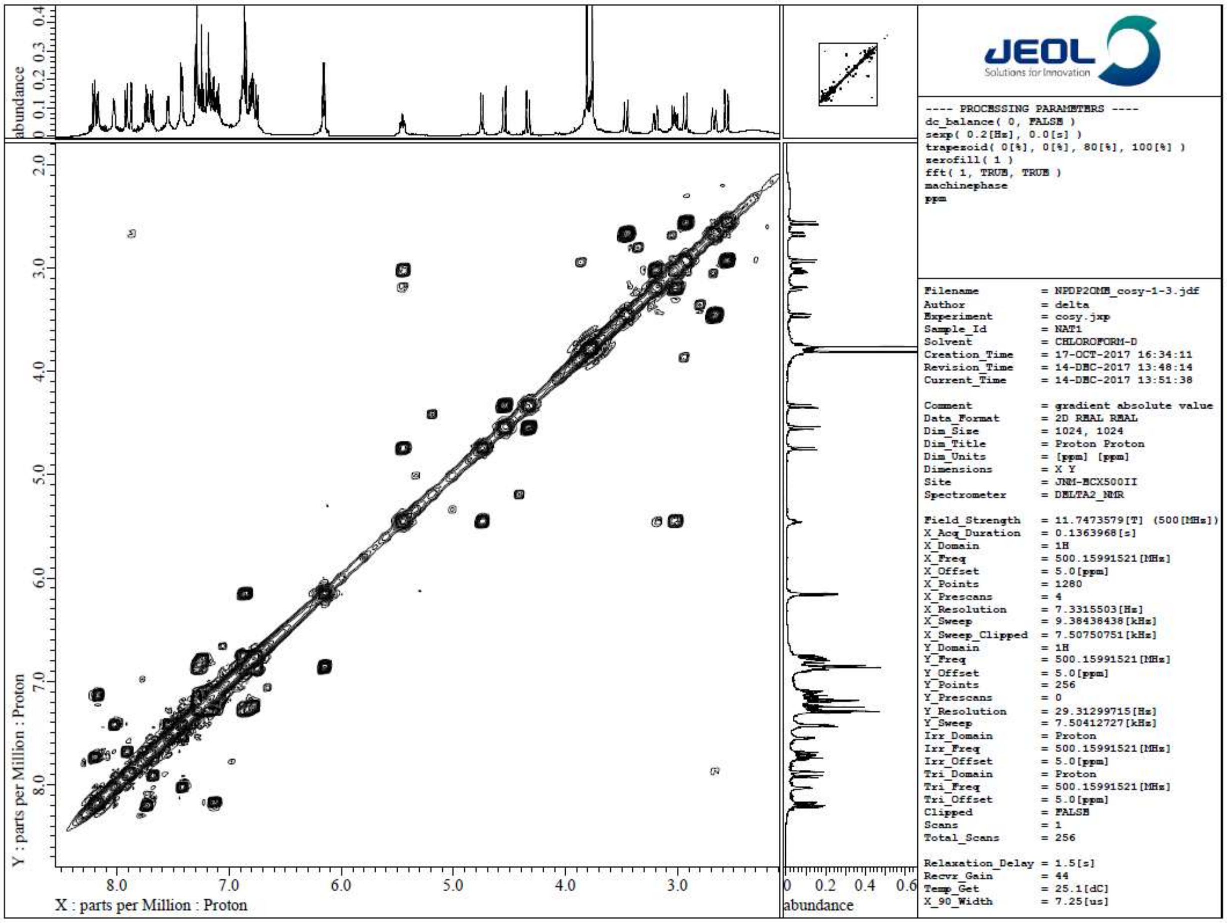

Figure S4. ^{1}H,^{1}H-COSY spectrum of **8j**

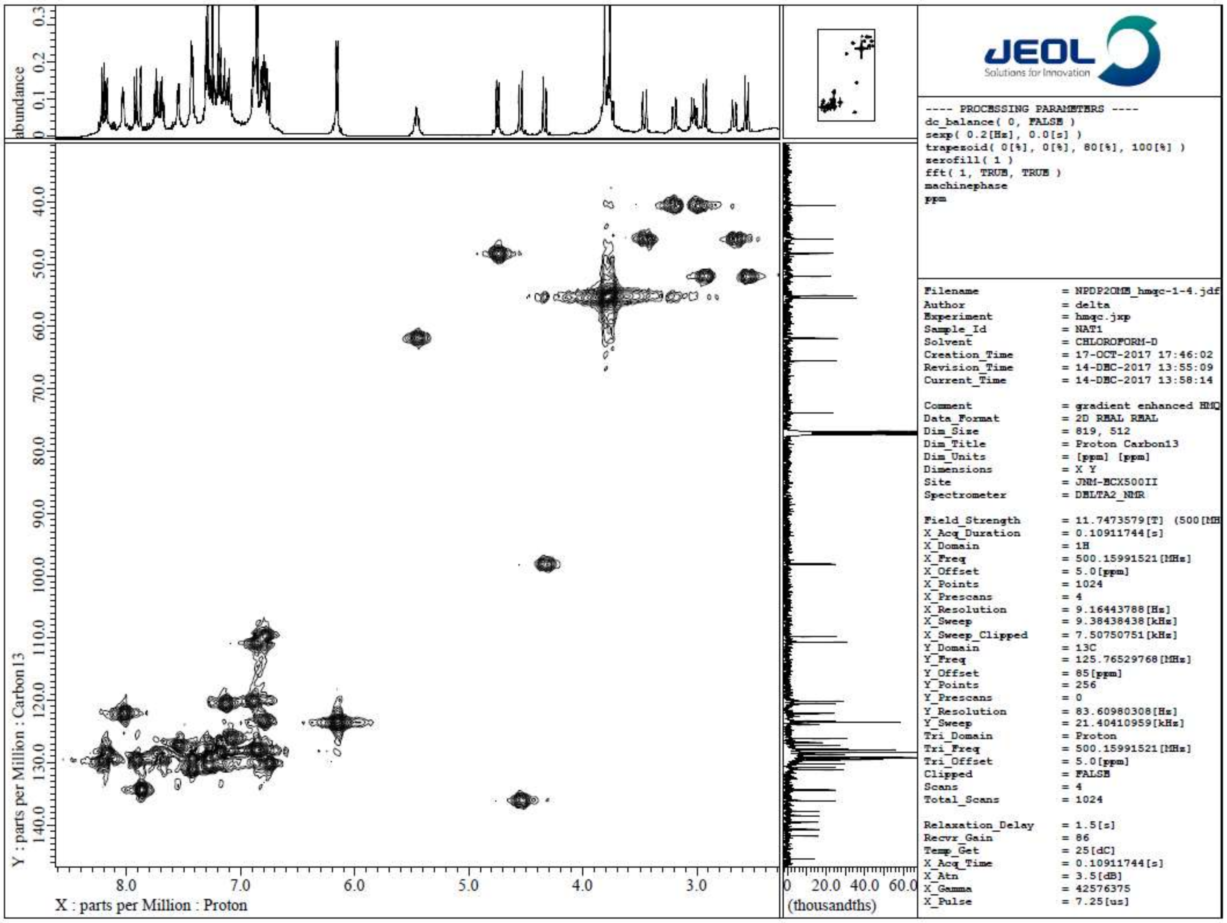

Figure S5. HMQC spectrum of **8j**

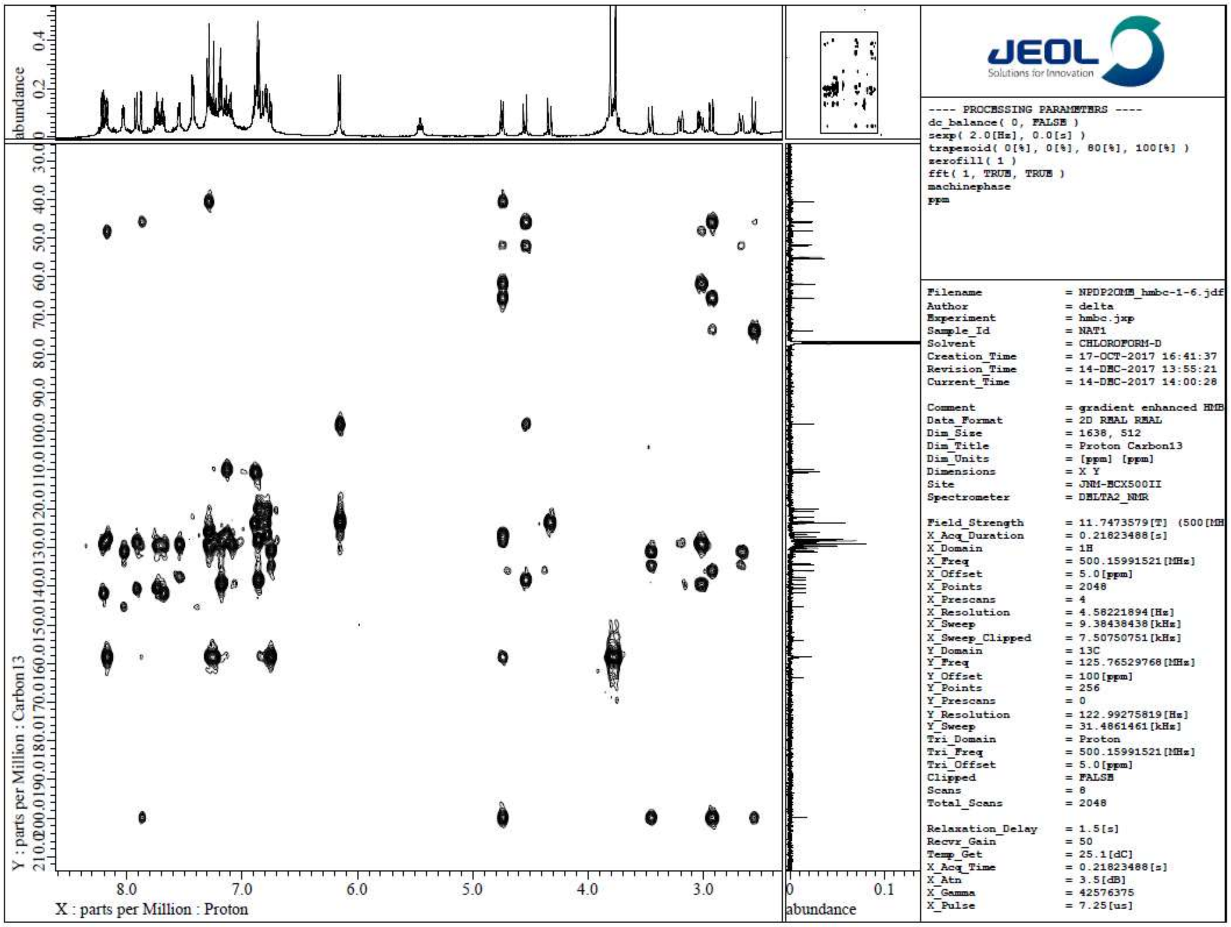

Figure S6. HMBC spectrum of **8j**

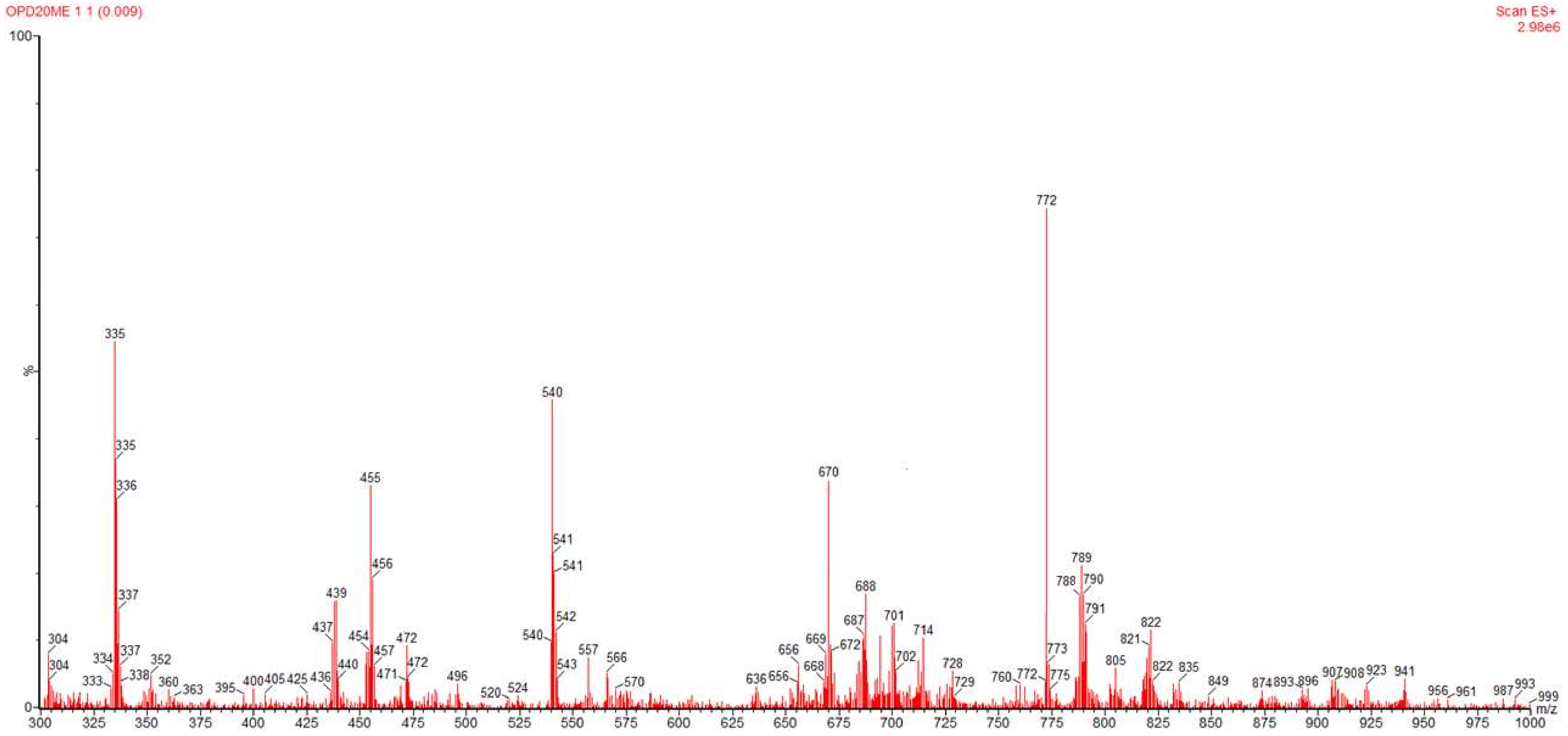

Figure S7. Mass spectrum of **8j**

2.2. Microbial Strain

Bacterial cultures used in the present studies were obtained from Microbial Type Culture Collection (MTCC), IMTECH, Chandigarh and American Type Culture Collection (ATCC) Manassas, USA. The bacterial strains were *Staphylococcus aureus* MTCC 96, *Staphylococcus epidermidis* MTCC 3615, *Bacillus subtilis* MTCC 441, *Escherichia coli* ATCC 25922, *Pseudomonas aeruginosa* ATCC 27584, *Klebsiella pneumoniae* MTCC 109, *Proteus vulgaris* ATCC 8427, *Proteus mirabilis* ATCC 7002, *Salmonella typhi* ATCC 19430, and *Salmonella paratyphi* MTCC 735.

The fungal cultures such as *Aspergillus niger*, *A. flavus*, *Candida albicans*, *Cryptococcus neoformans*, *Rhizopus* sp. were obtained from Bioline Laboratory, Coimbatore, Tamil Nadu. All the cultures were periodically sub-cultured and maintained with potato dextrose agar (PDA).

2.3. Preparation of microbial inoculum

One loop of bacterial inoculum was taken from a pure culture of the respective bacteria grown on slants and inoculated into 10 ml of nutrient broth. The broth suspension was then incubated at 37° C for 8 hrs to 12 hrs. The growth so obtained was used as inoculum for the sensitivity bioassay.

The filamentous fungi were grown on Sabouraud Dextrose Agar (SDA) slants at 25° C for 5 days and the spores were collected using sterile doubled distilled water and homogenized. Yeast was grown on Sabouraud Dextrose Broth (SDB) at 30° C for 24 h.

2.4. Antibiotic sensitivity test

The resistant pattern of different standard antibiotics was determined by disk diffusion methods (Bauer *et al*., 1966). The Mueller–Hinton agar (MHA) medium plates were inoculated with 0.1 ml of bacterial suspension grown in nutrient broth for 12 hrs at 37°C. Standard commercial antibiotic discs were used for testing the sensitivities of bacterial pathogens. Inoculated plates with antibiotic discs were incubated for 24 hrs and the diameter of resultant zone of inhibition was measured.

2.5. Antimicrobial activity of Dispiropyrrolidine derivatives

Antibacterial activity of Dispiropyrrolidines derivatives was tested by agar diffusion method (Bonev *et al.*, 2008). The plates containing MHA medium plates were spread with either 0.1 ml of the respective bacterial and fungal pathogens. Wells (6 mm in diameter) were cut from agar plates using a sterilized stainless steel borer and the wells were filled with 15, 25 and 50 µl of the Dispiropyrrolidines compounds (8a-8k). Streptomycin (30 µg), and DMSO was used as positive and negative control. The bacterial culture and fungal culture plates were incubated at 37° C and 25° C for 24 hrs and 3 days respectively. Further, the diameter of zone of inhibition was measured after incubation.

2.6. Determination of minimum inhibitory concentration of dispiropyrrolidine compound 8h

Minimum inhibitory concentration (MIC) of the dispiropyrrolidine compound 8h was testified by broth micro dilution technique. Briefly, the synthesized dispiropyrrolidine compound 8h was dissolved with DMSO and sterile distilled water (20% : 80%). The MIC was performed in 96 well plate contains the capacity of 300 µl volume. The suspension mixture contained, approximately 185 µl of nutrient broth, 10 µl of the dispiropyrrolidine compound 8h and 5 µl of the mid log phase Gram positive and Gram negative bacterial pathogens. Before, adding the bacterial pathogens, the suspension mixture was mixed thoroughly and diluted two-fold in each well. Finally, 5 µl of the bacterial cells were added to the well and mixed well for proper diffusion of the compound. Later, the 96 well plate was incubated at 37° C for 17 h. Standard streptomycin was used as the positive control. After incubation, the plate was visualized for observing the growth of the bacterial pathogens. The experiment was repeated three times.

2.7. Synergistic activity

Synergistic combinations were prepared with compound 8h and the antibiotics commonly resistant to bacterial pathogens. The concentrations of the compound 8h and antibiotics were began with their MIC value and then serially diluted into twofold. The efficient combinations were evaluated by calculating the fractional inhibitory concentration index (FICI) of each combination. The synergistic activity experiments were performed in triplicate.

FIC of compound 8h = MIC of compound 8h in combination with antibiotic/MIC of compound 8h alone

FIC of antibiotic = MIC of antibiotic in combination with compound 8h/MIC of antibiotic alone

FICI = FIC of compound 8h+FIC of antibiotic

Synergy activity was defined as an FICI ≤ 0.5. The FICI between 0.5 and 4.0 denotes that there is no interaction between the agents. If FIC > 4.0 indicates that there is antagonism between the two agents (Odds, 2003).

Table S1. Antibacterial activity of dispiropyrrolidine integrated indeno[1,2-*b*]quinoxaline heterocycli c hybrids **8a-k** against Gram positive bacterial pathogens

S.No.	Compounds	Zone of inhibition (mm) against Gram positive bacterial pathogens								
		S. aureus MTCC 96			*S. epidermidis* MTCC 3615			*B. subtilis* MTCC 441		
	Concentration (µg)	**15**	**25**	**50**	**15**	**25**	**50**	**15**	**25**	**50**
1	**8a**	13	17	20	0	17	23	14	22	24
2	**8b**	10	11	20	0	12	17	11	20	24
3	**8c**	11	18	23	0	15	21	15	20	22
4	**8d**	0	11	21	16	20	27	0	10	15
5	**8e**	10	12	15	0	15	22	19	21	24
6	**8f**	0	0	17	15	20	25	0	10	17

7	**8g**	0	11	19	0	14	20	18	24	23
8	**8h**	10	11	16	19	22	27	20	24	26
9	**8i**	0	11	16	14	18	20	13	22	24
10	**8j**	0	11	15	13	17	20	13	20	23
11	**8k**	11	15	20	16	20	24	18	22	25
14	Streptomycin (Positive Control)		25			23			20	
15	DMSO (Negative Control)		0			0			0	

Table S2. Antibacterial activity of dispiropyrrolidine integrated indeno[1,2-*b*]quinoxaline heterocycli c hybrids 8a-k against Gram negative bacterial pathogens

Name of the compound	Zone of inhibition (mm) against Gram negative bacterial pathogens																				
	E. coli ATCC 25922			*P. aeruginosa* ATCC 27584			*K. pneumoniae* MTCC 109			*P. vulgaris* ATCC 8427			*P. mirabilis* ATCC 7002			*S. typhi* ATCC 19430			*S. paratyphi* MTCC 735		
Conc (μg)	**15**	**25**	**50**	**15**	**25**	**50**	**15**	**25**	**50**	**15**	**25**	**50**	**15**	**25**	**50**	**15**	**25**	**50**	**15**	**25**	**50**
**8a**	9	12	18	0	0	0	0	9	14	14	16	19	0	0	9	0	10	15	0	11	17
**8b**	0	0	13	0	0	0	0	0	0	13	15	17	0	0	9	0	10	17	0	9	16
**8c**	0	0	12	0	11	11	0	11	15	11	13	16	0	0	9	0	11	16	0	11	18
**8d**	0	0	10	0	0	0	0	0	12	11	14	17	0	0	10	0	13	18	0	12	17
**8e**	0	0	10	0	0	0	0	0	14	12	16	18	9	11	13	10	11	17	0	10	18
**8f**	0	0	11	0	0	0	0	0	12	0	9	13	0	0	9	0	12	19	0	11	20
**8g**	0	11	9	0	0	10	0	0	0	14	17	19	0	0	15	9	14	17	0	12	19
**8h**	9	12	21	0	0	11	12	16	25	12	15	18	0	0	15	10	15	21	11	19	25
**8i**	0	10	14	0	9	13	0	0	9	14	16	19	0	0	11	0	0	17	0	11	19
**8j**	0	9	19	0	0	0	0	0	0	13	15	18	0	0	13	0	11	15	0	12	17
**8k**	0	10	20	0	0	0	0	0	12	12	16	18	0	0	0	9	12	17	0	10	18
Streptomycin (Positive Control)		25			20			16			21			14			22			25	

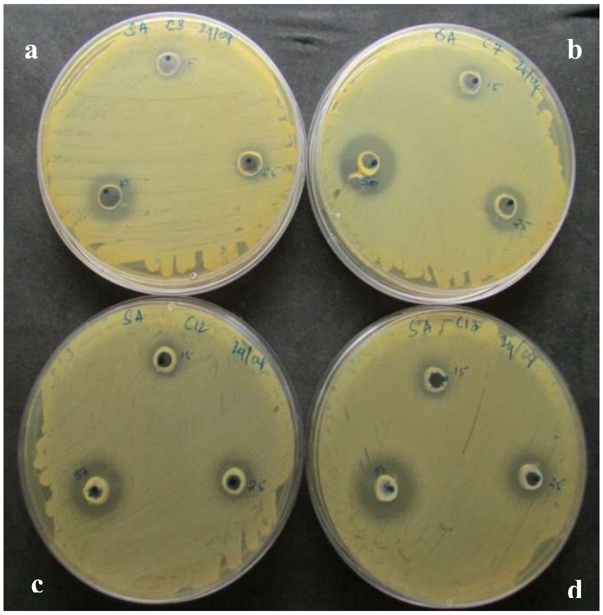

Figure S8. Antibacterial activity of Dispiropyrrolidines (8h, 8k, 8l, 8i) against *Staphylococcus aureus* MTCC 96; a) 8h, b) 8k, c) 8l, d) 8i

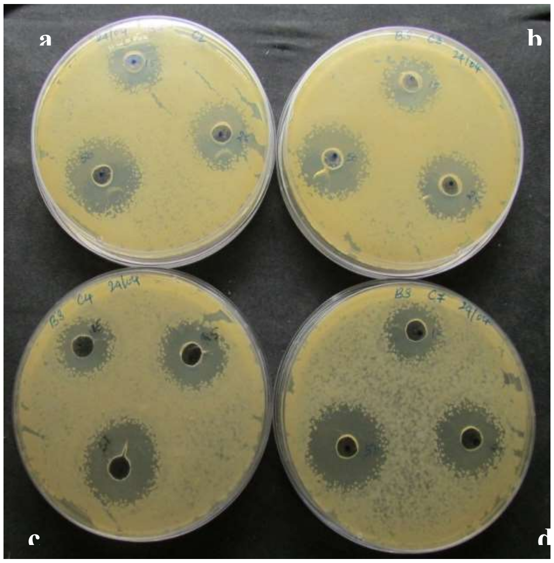

Figure S9. Antibacterial activity of Dispiropyrrolidines (8h, 8i, 8l, 8k) against *Bacillus subtilis* MTCC 441; a) 8h, b) 8i c) 8l, d) 8k

Table S3. Antifungal activity of Dispiropyrrolidines derivatives

S.No.	Compounds	Zone of inhibition (mm) / 50 µg				
		C. albicans BL 0142	*C. neoformans* BL 1703	*A. niger* BL 4217	*A. flavus* BL 5064	*Rhizopus* sp. BL 3389
1	**8a**	14.0	15.0	17.0	14.0	17.0
2	**8b**	0	16.0	14.0	13.0	15.0
3	**8c**	18.0	15.0	17.0	11.0	11.0
4	**8d**	9.0	9.5	0	0	0
5	**8e**	15.0	13.0	14.0	16.5	15.0
6	**8f**	17.0	16.0	15.0	15.0	16.0
7	**8g**	12.0	14.0	10.0	8.0	0
8	**8h**	17.0	18.0	15.5	16.0	17.0
9	**8i**	8.0	0	0	0	10.0
10	**8j**	0	14.0	15.0	13.0	0
12	**8k**	16.0	16.0	15.0	12.0	15.0
14	Nystatin (15 µg)	21.0	24.0	25.0	22.0	20.0
15	DMSO	0	0	0	0	0
